# Acquired activated protein C resistance and thrombosis in multiple myeloma patients

**DOI:** 10.1186/1477-9560-4-11

**Published:** 2006-08-21

**Authors:** Jiménez-Zepeda Víctor Hugo, Domínguez-Martínez Virginia Jeanet

**Affiliations:** 1 Department of Hematology and Oncology, Instituto Nacional de Ciencias Médicas y Nutrición Salvador Zubirán, Vasco de Quiroga 15, Colonia Sección XVI, Tlalpan, CP 14,000, DF, México,

## Abstract

**Background:**

An increased incidence of deep venous thrombosis (DVT) has been described in multiple myeloma (MM). A recently described mechanism of hypercoagulability in cancer patients including MM patients is acquired activated protein C resistance (APC-R). The purpose of the present study was to examine the association between the combination of thalidomide plus chemotherapy and DVT development in a cohort of patients with newly diagnosed multiple myeloma. We also evaluated the association between acquired activated protein C resistance and DVT.

**Methods:**

Patients with newly diagnosed and symptomatic MM (untreated or with one cycle of preceding chemotherapy) were evaluated. The present study is a prospective, descriptive, longitudinal and observational one. The coagulations tests were performed including: prothrombin time, activated partial thromboplastic time (aPTT), fibrinogen, anticardiolipin antibodies, lupus anticoagulant, antithrombin, protein C and protein S activities, factor VIII, activated protein C (APC) resistance, factor V Leiden, and quantitative D-dimers. Factor V Leiden mutation was detected by analysis of the polymerase chain reaction amplification of genomic DNA.

**Results:**

Fifty newly diagnosed multiple myeloma patients were included in the study. DVT was developed in 8 patients (16%). Six patients were confirmed to have acquired activated C protein resistance. All of them were tested twice. Four out of 6 patients developed DVT (66%), all of them received thalidomide at a median dose of 200 mg qd.

**Conclusion:**

APC-R appears to be a transitional condition that may be related to myeloma status. Thrombotic complications can affect morbidity and even mortality in these patients. To fully evaluate the potential synergistic anticancer activity of combinations of chemotherapy and thalidomide, effective prophylactic anticoagulation should be implemented in all controlled trials, at least during the first few cycles of treatment.

## Background

Recent reports of an increased incidence of venous thromboembolic events (VTE) in patients with multiple myeloma (MM) have sparked interest in hypercoagulability associated with hematologic malignancies and immunomodulator therapy [1]. A recently described mechanism of hypercoagulability in cancer patients including MM patients is acquired activated protein C resistance (APC-R) [2]. The fact that APC-R is not secondary to factor V Leiden has been described in up to 8% of all APC-R patients with aetiologies including oral contraceptives, pregnancy, anti-prothrombin antibodies, lupus anticoagulants, anti-phosphatidyl-ethanolamine antibodies and anti-protein S antibodies. APC-R remains an independent risk factor for VTE, regardless of aetiology [3].

With the increasing use of thalidomide as initial therapy for MM, deep venous thrombosis (DVT) and other thrombotic events also have emerged as major adverse events. Interestingly, Zangari et al (2002) reported that thalidomide therapy increased the risk of VTE to 50% in those with APC-R. In a peculiar manner, the increased risk of thrombosis in patients with MM is almost non existent when thalidomide is used as a single agent, but risk increases substantially when the drug is combined with high-dose corticosteroids or certain chemotherapy drugs [4]. The incremental risk suggests that the risk of DVT may be related to the interaction between drugs and their collective effect on malignant cells and some others events such as APC-R or the vascular endothelium [5].

The purpose of the present study was to examine the association between the combination of thalidomide plus chemotherapy and DVT development in a cohort of patients with newly diagnosed multiple myeloma. We also evaluated the association between acquired activated protein C resistance and DVT.

## Methods

Patients with newly diagnosed MM were evaluated. We enrolled all patients who fulfilled entire criteria for multiple myeloma during the period between January 1998 and December 2005. The present study is a prospective, descriptive, longitudinal and observational one. We collected clinical data and biochemical parameters at diagnosis and during their monitoring as inpatients and outpatients. Clinical features included age, sex, performance status, bone pain lesions, hepato-splenomegaly and plasmacytomas. Biochemical parameters data were collected including blood count, liver function test, blood chemistry, LDH, reactive C protein, B2-microglobulin, urine studies (urea, Bence Jones Proteinuria and light chains) and protein electrophoresis. Bone marrow trephine biopsy was performed and immunostaining also was included. Cytogenetics by karyotyping was evaluated. Performance status and bone lesions were scored according to previously described criteria. In addition, patients were grouped into clinical stages according to Durie-Salmon criteria. Imaging studies where performed (bone series, CT scan and MRI) when necessary.

### Treatment schemes

Thalidomide was prescribed in an oral dose of 100 mg qhs and increased by 50 mg every 7 days to a maximum dose of 300 mg, depending on side effects. Dexamethasone was given in an oral dose of 20 mg/m2 each morning after breakfast on days 1–4, 9–12 and 17–20, followed by 10 days without therapy prior to the next cycle. VAD was given as an out-patient regimen including: vincristine (0.4 mg/day, continuous IV), doxorubicin (9 mg/m2/day, continuous IV) and dexamethasone (40 mg/day PO) with a median of 6 courses. Melphalan and prednisolone were prescribed in 4 days courses (M 6–9 mg/m2/day PO and P 100 mg/day PO) at 4–6 week intervals.

### Response and toxicity criteria

Response was assessed according to the European, International, and Autologous Bone Marrow Transplant Registries (EBMT/IBMTR/ABMTR) criteria [6]. Thus, responses were categorized as CR (negative immunofixation, <5% bone marrow plasma cells, disappearance of soft tissue plasmacytomas), partial response (PR) (decrease in the M-protein > or equal to 50%, decrease in the light chain urine protein excretion > or equal to 90% plus reduction > or equal to 50%, in the size of soft tissue plasmacytomas), or minimal response (MR) (25 to 49% decrease in the serum M-component, 50–89% reduction in the urine light chain protein excretion and 25 to 49% decrease in the size of soft tissue plasmacytomas). The results were analyzed on an intention-to-treat basis since all patients who started treatment were included in the analysis.

### Coagulations tests and thrombosis

Citrate plasma was used to investigate coagulation and anticoagulation parameters. The coagulations tests were performed including: prothrombin time, activated partial thromboplastic time (aPTT), fibrinogen, anticardiolipin antibodies, lupus anticoagulant, antithrombin III, protein C and protein S activities, activated protein C (APC) resistance, Factor V Leiden, and quantitative D-dimers. Factor V Leiden mutation was detected by analysis of the polymerase chain reaction amplification of genomic DNA. The DNA was extracted from ethylenediamine tetraacetic acid anticoagulated blood. APC resistance was tested using fresh or frozen plasma stored at -70°C. The aPTT was measured using a compact coagulometer. The APC resistance was determined on citrated plasma using an aPTT-based Food and Drug Administration-approved resistance assay with factor V deficient plasma (Chromogenix Instrumentation Laboratory, Milano, Italy). The ratios between the aPTT with or without the presence of activated protein C were calculated. The patient was considered positive if the ratio was less than 2. Samples that were reported positive for APC-R were retested using both, second generation assay and the original assay (Dade, Behring and Chromogenix) to confirm the previous report.

MM patients were regularly evaluated by the medical staff and underwent Doppler ultrasound examination if there were clinical signs or symptoms suggestive of DVT. The date of DVT was defined as the date of its confirmation by Doppler ultrasonography, which was performed on all patients with clinical indication of DVT.

### Statistical analysis

was performed using SPSS version 13.0. Fisher's exact test was used to evaluate the statistical significance of associations in two-way contingency tables. Cochran-Mantel-Haenszel methods were used to evaluate the association between APC resistance and DVT occurrence while controlling for thalidomide exposure. A p value of less than 0.05 was considered as statistically significant.

## Results

Fifty newly diagnosed multiple myeloma patients were included in the study, 27 (54%) male and 23 (46%) female, aged 45–83 (mean 63). Underlying diagnosis was IgG MM in 40 (80%), IgA in 8 (16%) and light chain disease in 2 (4%). Table 1

**Table 1:** Clinical characteristics

Gender	Male	27 (54%)
	Female	23 (46%)
*Age	63 (45–83 years)	
*Marrow plasma cells (%)	48% (23–90%)	
Immunoglobulins	IgG	40 (80%)
	IgA	8 (16%)
	LC	2 (4%)
*Bone lesions	75%	
*Creatinine mg/dl	1.5 (0.6–5.6)	
*Hemoglobin (g/dl)	11.8 (8.2–14.9)	
Plasmacytoma	9 (18%)	
*B2 microglobulin	4.8 (1.2–9.6)	
*Albumin g/L	34.9 (21.5–46)	
APC-R	6 (12%)	

*Levels are expressed as median values

Median age at diagnosis was 63 years (45–83). Median plasma cells number in marrow was 48% (23–90%). Seventy five percent of patients presented bone lesions at diagnosis. Plasmacytoma was seen in 9 patients, 2 with extramedullary plasmacytoma. Median hemoglobin was 11.82 g/dl (8.2–14.9). B2 microglobulin was observed with a median of 4.82 mg/L (1.2–9.6) and albumin level expressed a median value of 3.49 g/L (2.15–4.60). Clinical characteristics are seen in Table 1. According to Durie-Salmon criteria patients were grouped into: I A (n 7, 14%), I B (n 4, 8%), IIA (n 13, 26%), II B (n 5, 10%), III A (n 14, 28%) and III B (n 7, 14%). Table 2

**Table 2:** Stage according to Durie-Salmon

**Stage**	**N**	**%**
IA	7	14
IB	4	8
IIA	13	26
IIB	5	10
IIIA	14	28
IIIB	7	14

Patients received as frontline therapy three different schemes of chemotherapy. VAD scheme was given in 20 patients (40%) while thalidomide and dexamethasone was given in 48% (24 patients). Only 6 patients (12%) received melphalan and prednisolone. Table 3 Median follow-up was 42 months. After 6 cycles, patients receiving thalidomide plus dexamethasone showed an overall objective response rate of 75% while VAD group only reached 55% (CR, NCR and PR) p0.005.

**Table 3:** Overall objective response according to scheme of therapy

**Scheme**	**N**	**%**
**VAD**	**20**	**40**
Overall objective response	11/20	55
**Thalidomide plus dexamethasone**	**24**	**48**
Overall objective response	18/24	75
**Melphalan plus prednisone**	**6**	**12**
Overall objective response	2/6	33

DVT was developed in 8 patients (16%). Known risk factors for DVT, such as central venous catheters (CVC) (present in 25% patients, none developed DVT), performance status, and hormonal therapy were not significantly different between these 2 groups of chemotherapy (all p values > 0.2). None patient has prior history of DVT and neither one has been receiving anticoagulation at the time of enrollment. DVT was developed in 5 patients treated with thalidomide plus dexamethasone, 1 patient treated with VAD and thalidomide and 2 more receiving VAD only. A significant shorter time to DVT was observed in patients exposed to VAD chemotherapy (first 2 cycles p = to 0.007). Table 4 Seven patients developed DVT in the lower extremities and the remainder developed DVT and pulmonary embolism.

**Table 4:** Time to thrombosis according to therapy

**Patient**	**Therapy**	**Time to thrombosis Months**	**APC-R Yes/Not**
1	TD	6	Yes
2	TD	7	Yes
3	TD	8	Yes
4	TD	6	Yes
5	TD	6	Not
6	VAD-T	3	Not
7	VAD	2	Not
8	VAD	2	Not

**p value 0.007 time to thrombosis between TD and VAD therapy**.

TD: Thalidomide plus dexamethasone, VAD; Vincristine, Doxorubicin and Dexamethasone; VAD-T: VAD plus thalidomide; APC-R Acquired activated protein C resistance.

Six patients were confirmed to have activated C protein resistance. Lupus anticoagulant was negative in all cases. The ratios observed in this study for APCR are shown in Table 5. Factor VIII levels may also influence the result of APCR. Since frequent and important elevated levels of factor VIII in MM are described, the level of factor VIII concomitant to APCR determination seems to be mandatory, we found normal levels of factor VIII for patients with APCR. In parallel, we measured D-dimers, those are shown in Table 5. Patients positive for APC resistance were re-tested using a second generation assay and the original assay (Dade-Behring), we found a 100% concordance between both methods. Of these 6 patients four developed DVT (66%), all of the cases received thalidomide at a median dose of 200 mg qd. Two patients with APC-R did not present DVT. All cases developed the thrombosis event during the first year of treatment, particularly during the induction phase in the VAD group (time to thrombosis of 2.3 months versus 6.6 months for thalidomide plus dexamethasone). After patients achieved any type of response according to EBMT/IBMTR/ABMTR criteria, another re-test of ACP-R was performed; it was negative in 7/8. All patients received anticoagulation therapy based on coumadin at a target INR of 2.5–3.0. Neither one developed re-thrombosis but one patient developed secondary plasma cell leukemia. Patients who were positive for ACP-R were tested for factor V mutation but only one was positive for this abnormality. The patient with genetic activated protein C resistance did not present DVT. We did not find abnormalities in protein S and C levels at our patients.

**Table 5:** Coagulation tests for newly diagnosed patients with multiple myeloma

**Test**	**Result**
TVE	8/50 (16%)
TVE and APC-R	4/6 (66%)
APC-R	6/50 (12%)
Median ratio APC-R	6/50 1.5 (1.4–1.6)
*Factor VIII (iu/dl)	96 iu/dl (n 50)
*D-dimer (ng/ml)	456 ng/ml (n 50)
*a PTT (seconds)	29 s (n 50)
*PT (seconds)	13 s (n 50)
Antithrombin (u/dl)	99 u/dl (n 50)
Factor V Mutation	1/50 (2%)
Lupus anticoagulant	0/50
*Protein S (u/dl)	98 u/dl (n 50)
*Protein C (u/dl)	96 u/dl (n 50)

TVE Thromboembolic Events, APC-R Acquired Activated Protein C Resistance. * Levels are expressed as median values

## Discussion

High cumulative risk of thrombotic events in myeloma patients has been already reported [7]. The present study confirmed that myeloma patients treated with thalidomide and multi-agent chemotherapy are at increased risk of DVT. Many DVT risk factors have been identified. Alikhan et al, (2003) reported many of them [8]. With the median follow-up of 42 months, the rate of DVT was 16% at our study. The thrombotic phenomenon occurred early in the course of treatment (100% during the first year at our patients). The percentage of DVT associated with treatment with thalidomide and chemotherapy including dexamethasone varies according to the series (4–28%), we found an overall incidence of 12% (6/50) of DVT in patients receiving thalidomide but when patients developed Acquired activated protein C resistance (ACP-R), the DVT incidence increases (66%).

The possible production of auto-antibodies against protein C in these patients could be the explanation for the transient APC resistance phenotype observed in some of the patients [9,10]. The manufacturer recommends 1/5 dilution for the APC-R test, we used this dilution. High titer of antibodies could still inhibit the activity of APC based on previous reports [10]. Monoclonal immunoglobulins have often an antibody activity (lupus anticoagulant or auto-antibodies against protein C have been described) or cryoglobulin properties. Lupus anticoagulant can induce false positive APCR ratio. A 1:40 dilution seems to be the optimal dilution to avoid lupus anticoagulant interference. We used 1:5 dilution, which is probably not sufficient to avoid interference of high titer antibodies against protein C. Search for lupus anticoagulant was important in this case. Patients with APCR were negative for lupus anticoagulant. We found only one patient with genetic PCR who did not develop thrombosis. People with the APC-R phenotype because on inherited or acquired conditions usually have APC ratios below 2. Predilution of samples with factor V deficient plasma provides 100% sensitivity for the factor V Leiden mutation and reduces the influence of plasma handling, heparin therapy and oral warfarin [11]. In order to confirm APC-R, samples were performed using the original assay and the second generation assay, we found a 100% concordance between both assays.

The activated protein C resistance (APCr) phenotype is found in around 40% of thrombophilic Mexican Mestizo individuals; since only very few display the factor V gene Leiden (Arg506Gln) mutation, it was considered of interest looking for other factor V gene mutations associated to thrombophilia: The HR2 haplotype, the factor V Cambridge (Arg306Thr), the factor V Hong Kong (Arg306Gly) and the FV Liverpool (Ile359Thr). In 39 individuals, the FV Leiden was found in 10%, the HR2 haplotype in 28%, the FV Hong Kong in 2%, whereas the FV Cambridge and FV Liverpool gene mutations were not found in any individual in a Mexican population [12].

There is a strong association between DVT and exposure to doxorubicin/thalidomide. The thrombotic mechanism seems to be multifactorial in the DVT patients [13]. The highest incidence of DVT is noticed during the first cycle of doxorubicin containing chemotherapy in some other series [14,15]. It supports the fact that tumor load and myeloma characteristics are not the restrictive factors associated to development of DVT. We also found better outcomes in those patients receiving thalidomide plus dexamethasone than VAD receivers according to some other reports [16-18]. In conclusion, it is clear that in multiple myeloma thalidomide increases the thrombotic risk, particularly in combination with chemotherapy and APC-R could be an additional factor associated to the DVT pathogenesis. APC-R appears to be a transitional condition may be related to myeloma status. Thrombotic complications can affect morbidity and even mortality in these patients. To fully evaluate the potential synergistic anticancer activity of combinations of chemotherapy and thalidomide, effective prophylactic anticoagulation should be implemented in all controlled trials, at least during the first few cycles of treatment.

## Competing interests

The author(s) declare that they have no competing interests.

## Authors' contributions

VJ participated designing the study, collecting data, reviewing and writing the article.

VD participated collecting data, reviewing and writing the article.

Both authors read and approved the final manuscript.

## Funding source

VJ and VD are Clinical Investigators of the INCMNSZ Research fund.
